# Smoking as an independent predictor of long term care certification: The Yamagata cohort study

**DOI:** 10.18332/tid/224865

**Published:** 2026-07-10

**Authors:** Yoko Matsunami, Masayoshi Souri, Ri Sho, Natsuko Suzuki, Midori Furuse, Tsuneo Konta

**Affiliations:** 1 School of Nursing, Faculty of Medicine, Yamagata University, Yamagata, Japan; 2 Department of Public Health and Hygiene, Yamagata University Graduate School of Medical Science, Yamagata, Japan

**Keywords:** smoking cessation, long-term care certification, cohort study, risk factors

## Abstract

**INTRODUCTION:**

Smoking has been associated with disease risk; however, whether smoking is an independent predictor of long-term care (LTC) certification remains uninvestigated. Therefore, this study aimed to examine the association between smoking status and LTC certification.

**METHODS:**

This prospective cohort study was conducted using data from the Yamagata cohort study in Japan. Participants were followed for a mean of 7.6 years (SD=1.8). Individuals with available baseline data on smoking status and relevant covariates were included. The main exposure was baseline smoking status (current, former, and never smokers), and the primary outcome was LTC certification incidence during follow-up. Associations between smoking status and LTC certification were evaluated using Cox proportional hazards models, with sequential adjustment for potential confounders, including demographic factors, lifestyle habits, and comorbidities.

**RESULTS:**

At baseline, the overall smoking prevalence rate was 12.4% (22.4% in men, 4.6% in women). At a mean follow-up of 7.6 years (SD=1.8), LTC certification was issued to 262 (2.6%) individuals: 137 men (3.0%), and 125 women (2.1%). Unadjusted analysis using a Cox proportional hazards model indicated a higher hazard ratio (HR) for current smokers (HR=1.44; 95% CI: 1.03–2.02), which remained significant after adjustment for sex and age (AHR=1.81; 95% CI: 1.23–2.66). This persisted even after adjusting for sex, age, physical activity, alcohol consumption, hypertension, diabetes, and dyslipidemia (AHR=1.83; 95% CI: 1.25–2.69), and after accounting for nine factors, including stroke and ischemic heart disease (AHR=1.86; 95% CI: 1.27–2.73). Never and former smokers showed no differences in adjusted models. A sensitivity analysis excluding patients with a history of cardiovascular disease yielded similar AHRs.

**CONCLUSIONS:**

Overall, current smokers had a higher risk of LTC certification than never smokers, suggesting that smoking may be an independent risk factor.

## INTRODUCTION

Japan faces a super-aged society with an increasing demand for medical and long-term care (LTC) among older adults^1^, coinciding with a declining younger population due to low birth rates, leading to rising care costs^2,3^. The Japanese government launched the long-term care insurance (LTCI) system in 2000 in response to population aging and increasing healthcare needs and expenditures^4^. The aims of the LTCI system were to shift the burden of family caregiving to society, distribute costs through insurance premiums, and integrate medical and welfare services^4^. Older people with a certification for LTCI service needs, can utilize facility services, in-home services, and community-based services depending on their physical and cognitive impairments^4^. After the implementation of the LTCI system, there was a rapid increase in persons certified for LTCI service needs, with a corresponding increase in financial burden on the government^4^. To tackle this issue, countermeasures necessitate detecting diseases early and extending healthy life expectancy through individual preventive actions and health-related behavioral changes. Extending healthy life expectancy has been widely recognized as an important public health objective and is associated with improved quality of life and reductions in healthcare and long-term care expenditures^2,3^.

Smoking has been established by numerous epidemiological studies to be a risk factor for various diseases that increase mortality. As highlighted in the US Surgeon General’s report, smoking contributes to the development of a wide range of diseases, including cancer, cardiovascular diseases (CVDs), and respiratory diseases^5^. In Japan, a comparative risk assessment study^6^ identified smoking and hypertension as two major preventable risk factors contributing to non-communicable diseases and injury-related deaths among Japanese individuals^6^. Similarly, the Japan collaborative cohort study demonstrated that smoking increased the risk of mortality from CVDs, such as stroke and ischemic heart disease^7,8^. Furthermore, previous studies showed that smoking clearly led to various lifestyle-related diseases, including increased risks of chronic obstructive pulmonary disease (COPD)^9^ and type 2 diabetes^10^.

Smoking is also associated with age-related health problems. With respect to dementia, smoking has been reported to be associated with cognitive decline, disease onset, and an increased risk of dementia^11,12^. In the musculoskeletal domain, meta-analyses showed that smoking was associated with reduced bone mineral density^13^ and fractures^14^. A follow-up study of NIPPON DATA80 reported that smoking in middle age increased the long-term risk of reduced activities of daily living^15^. A systematic review also identified smoking as a predictor of frailty onset^16^. A report by Japan’s Ministry of Health, Labor, and Welfare revealed that dementia, cerebrovascular disease (stroke), and fractures/falls were the primary causes of LTC certification^17^. Considering the association between smoking and these conditions, smoking cessation is crucial for preventing LTC certification. Previous studies conducted in Japan reported that smokers had shorter healthy life expectancy than never smokers^18^ and smoking was associated with higher sex- and age-adjusted hazard ratios (AHRs) and population-attributable risk fractions for loss of independence^19^.

Numerous epidemiological studies have documented the health benefits of smoking cessation in the general population. The Japan Public Health Center-based (JPHC) prospective study^20^, a cohort study involving Japanese individuals, reported a significantly higher overall mortality rate among smokers than among never smokers, confirming that smoking is a primary cause of cancer and CVDs. The JPHC study also clarified that the risk of mortality after smoking cessation decreased over time, ultimately declining to levels comparable to those of never smokers^20^. Furthermore, a large-scale international cohort study showed that the risk of CVD improved within 5 years of smoking cessation, and that the longer the period of abstinence, the greater the reduction in CVD risk^21^. Previous reports also consistently confirmed the cardiovascular risk-reducing effects of smoking cessation^22,23^. In particular, a randomized controlled trial^24^ involving smokers with mild obstructive pulmonary disease reported that the 15-year all-cause mortality rate in the smoking cessation intervention group was 15% lower than that in the control group and that the mortality rate from respiratory diseases (excluding lung cancer) was approximately 50% lower. Taken together, these findings suggest that smoking cessation may contribute to extending healthy life expectancy and reducing the risk of LTC certification.

Although smoking has been linked to various adverse health outcomes, including mortality and functional decline, its role in the progression to LTC requires further investigation. Evidence on whether smoking independently affects the risk of LTC certification, accounting for potential confounding factors, remains limited.

Accordingly, this study aimed to examine the longitudinal association between current smoking status and the incidence of LTC certification in a Japanese cohort.

## METHODS

### Study design and setting

This study was designed as a population-based prospective cohort study conducted as part of the Yamagata cohort study: a contemporary genome cohort to elucidate the relationship between environmental and genetic factors. STROBE checklist is given in the Supplementary file. The project was established under the Global Center of Excellence Program at Yamagata University.

Participants were recruited from residents of Yamagata prefecture who underwent specific health checkups between January 2010 and December 2015 and were followed through December 2020.

Participants were recruited from individuals undergoing routine municipal health checkups; therefore, the sample can be considered a community-based convenience sample of health checkup attendees, rather than a random or strictly population-representative sample.

### Study population and sampling

**Figure 1:**
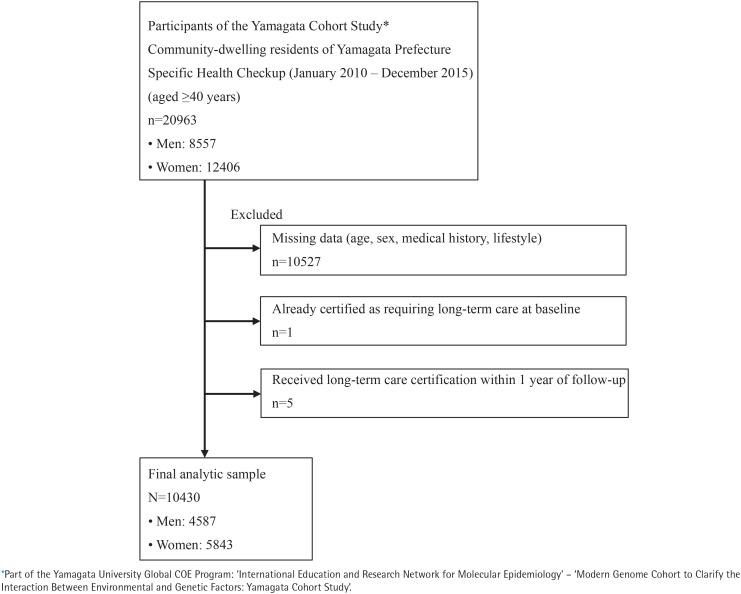
Flow diagram of participant selection

This study included 20963 adults (8557 men and 12406 women) aged ≥40 years who resided in Yamagata prefecture and underwent specific health checkups from January 2010 to December 2015. Among them, 10527 individuals with missing baseline data on covariates essential for adjustment (namely, smoking status, age, sex, physical activity, alcohol consumption, hypertension, diabetes, dyslipidemia, stroke, and ischemic heart disease) were excluded to ensure data completeness for a robust analysis. Additionally, individuals already certified as requiring LTC at baseline and those certified within 1 year of follow-up were excluded from the analyses. In this study, the incidence of newly certified LTC need was defined as the outcome. Therefore, individuals who had already been certified as requiring LTC at baseline were excluded from the analysis, as they had already experienced the event and were not appropriate for inclusion in the statistical analyses. Furthermore, individuals who were certified within 1 year after baseline were also excluded, because they may have already been in an undiagnosed state of LTC need or its progression at baseline. Ultimately, statistical analyses were conducted with 10430 participants (4587 men and 5843 women) (Figure 1).

### Ethics

Ethical approval was obtained from the Ethics Review Committee of Yamagata University (Approval number: 2023-295; Date: 7 February 2024). When inviting participants to take part in the present study, we provided an overview of the survey, including the voluntary nature of participation and the protection of personal information, and subsequently obtained written informed consent.

*Survey items*

At baseline, self-administered questionnaires covering medical history, alcohol consumption, smoking, and physical activity were mailed to the study participants during specified health checkups, and their responses were recorded. Smoking status and lifestyle-related variables were assessed only at baseline and were not updated during the follow-up period; therefore, potential changes over time could not be captured. Responses to questions were classified as follows: regarding a history of hypertension, diabetes, and dyslipidemia, clinical laboratory values, including blood pressure and blood values, were evaluated at the health checkup site.

*Smoking status*

Smoking status was assessed using a self-administered questionnaire and categorized into: current smoker, never smoker, and former smoker. It was assessed based on a self-administered questionnaire in which participants were asked about their current smoking behavior. Participants who reported that they were currently smoking were classified as current smokers, while those who reported that they had never smoked or had quit smoking were classified as never smokers and former smokers, respectively.

This classification was based on self-perceived status rather than a time-bound definition (e.g. smoking within the past 30 days), without a defined minimum lifetime exposure threshold or time since cessation, and no biochemical validation (such as cotinine measurement) was performed.

*Alcohol consumption*

Alcohol consumption status was categorized into two groups: current drinker and non-drinker. This category includes both lifetime abstainers and former drinkers, and these groups could not be distinguished due to data limitations (e.g. ‘sick quitter’ effect).

*Physical activity*

Physical activity status was categorized into two groups: engages in light-to-moderate physical activity (perspiration level) for at least 30 min per session, at least twice a week, for over 1 year; and does not engage in such activity.

*Hypertension*

Hypertension was defined based on OR criteria as any of the following: systolic blood pressure ≥140 mmHg, diastolic blood pressure ≥90 mmHg, or use of antihypertensive medication.

*Diabetes*

Diabetes was defined based on OR criteria as any of the following: fasting blood glucose ≥126 mg/dL, HbA1c ≥6.5% (NGSP: National Glycohemoglobin Standardization Program^25^), HbA1c ≥6.1% (Japan diabetes society standard^25^), or use of antidiabetic medication. The inclusion of both HbA1c thresholds reflects differences between Japanese and international diagnostic criteria.

*Dyslipidemia*

Dyslipidemia was defined based on OR criteria as any of the following: total cholesterol ≥220 mg/dL, triglycerides ≥150 mg/dL, use of lipid-lowering medication. LDL-C and HDL-C were not included due to data availability constraints, which may limit comparability with guideline-based definitions.

*Stroke*

A history of stroke was defined based on self-reported questionnaire responses indicating a physician diagnosis of stroke or current treatment for stroke.

*Ischemic heart disease*

A history of ischemic heart disease was defined based on self-reported physician diagnosis or treatment history of myocardial infarction or angina pectoris. These conditions were analyzed together as ischemic heart disease, although they differ in diagnostic certainty and clinical severity.

### Outcome definition: incident LTC

The outcome was incident LTC certification, identified using municipal LTCI databases. The date of LTC certification was used as the event date. For the analysis of the association with LTC incidence, the study participants were followed until the end of 2020 (maximum follow-up period of 10 years), and information on incident LTC date and severity based on LTC registration was obtained. Data on this incidence of LTC were obtained from municipal public LTCI system databases, with those who were newly certified as eligible for the LTCI benefit considered as LTC incidence.

In Japan’s LTCI system, eligibility is assessed using a 74-item questionnaire based on activities of daily living, followed by a decision made by a long-term care approval board based on the initial computer decision, the home-visit report, and a medical doctor’s opinion^4^. There are seven levels of long-term care that require certificates: support levels 1 and 2 and care need levels 1 (least disabled) to 5 (most disabled)^4^. In this study, LTC was defined as care level 1 or higher in the Japanese LTCI system, representing individuals with clinically meaningful functional impairment requiring assistance with daily activities. In Japan’s LTCI system, support levels generally indicate milder functional limitations and partial or preventive support needs, whereas care-need levels indicate more substantial needs for assistance with daily living^4^. Therefore, focusing on care-need levels 1–5 allowed us to target individuals with clearer long-term care needs and clinically meaningful ADL limitations.

### Follow-up

Participants were registered at the date of LTC certification (event), the end of follow-up (December 2020), or loss to follow-up (if applicable). Competing risks such as death were not explicitly modeled, which may affect estimates.

Smoking and lifestyle variables were assessed only at baseline and were not updated during follow-up, meaning changes in exposure over time were not captured.

### Statistical analysis

The association between the participants’ smoking status at baseline and their basic characteristics and lifestyle habits was first analyzed. Continuous variables are expressed as means and standard deviations (SDs), and categorical variables as frequencies and percentages. Means and proportions were compared using analysis of variance and the chi-squared test, respectively.

Subsequently, the association between LTC certification as the endpoint and smoking status as the explanatory variable was examined using unadjusted and multivariate-adjusted Cox proportional hazards models adjusted for background factors. The proportional hazards assumption was evaluated graphically. No apparent violation of proportionality was identified. Multivariate adjustment included nine factors: sex, age, regular physical activity (≥30 min), alcohol consumption, hypertension, diabetes, dyslipidemia, stroke, and ischemic heart disease. Analyses for the entire cohort were conducted, followed by separate analyses for men and women.

Considering that individuals with a history of stroke or ischemic heart disease at baseline may have severe functional impairment requiring LTC certification or experience accelerated disease progression, potentially confounding the association with smoking, a sensitivity analysis that excluded participants with a history of CVD (stroke or ischemic heart disease) at baseline was performed to assess the robustness of the association after excluding the influence of existing CVDs.

All statistical analyses were performed using JMP^®^ 18 (JMP Statistical Discovery LLC, Cary, NC, USA), with statistical significance set at p<0.05.

## RESULTS

At baseline, the overall smoking prevalence rate was 12.4% (22.4% in men and 4.6% in women). Current, former, and never smokers differed with respect to sex, age, physical activity, alcohol consumption, and the presence of hypertension, diabetes, dyslipidemia, stroke, and ischemic heart disease (Table 1). At a mean follow-up period of 7.6 years (SD=1.8), LTC certification was issued to 262 (2.6) individuals: 137 men (3.0%) and 125 women 2.1%).

**Table 1:** Baseline demographic, lifestyle, and clinical characteristics according to smoking status in the Yamagata cohort study, Yamagata, Japan, baseline assessment period January 2010–December 2015 (N=10430)

*Characteristics*	*Total* *n (%)*	*Current smokers* *n (%)*	*Never smokers* *n (%)*	*Former smokers* *n (%)*	p*
Total, n	10430	1291	6700	2439	
Men	4587 (44.0)	1025 (22.4)	1490 (32.5)	2072 (45.2)	<0.01
Women	5843 (56.0)	266 (4.6)	5210 (89.2)	367 (6.3)	
Age (years), mean (SD)	63.5 (7.9)	60.7 (9.2)	63.8 (7.7)	64.3 (8.9)	<0.01
Physical activity	4997 (47.9)	497 (38.5)	3232 (48.2)	1268 (52.0)	<0.01
Alcohol consumption	4960 (47.6)	920 (71.3)	2293 (34.2)	1747 (71.6)	<0.01
Hypertension	4848 (46.5)	503 (39.0)	2993 (44.7)	1352 (55.4)	<0.01
Diabetes	1192 (11.4)	154 (11.9)	646 (9.6)	392 (16.1)	<0.01
Dyslipidemia	5755 (55.2)	672 (52.1)	3748 (55.9)	1335 (54.7)	0.03
Stroke	218 (2.1)	19 (1.5)	107 (1.6)	92 (3.8)	<0.01
Ischemic heart disease	172 (1.7)	12 (0.9)	76 (1.1)	84 (3.4)	<0.01

P-values by mean age: analysis of variance, others: chi-squared test.

* Statistically significant at p<0.05.

The results of the Cox proportional hazards model analysis for the association between smoking status and LTC certification are presented in Table 2. The unadjusted analysis using a Cox proportional hazards model indicated a higher HR for current smokers (HR=1.44; 95% CI: 1.03–2.02), which remained significant after adjustment for sex and age (AHR=1.81; 95% CI: 1.23–2.66). This result persisted even after adjusting for sex, age, physical activity, alcohol consumption, hypertension, diabetes, and dyslipidemia (AHR=1.83; 95% CI: 1.25–2.69), and after accounting for nine factors, including stroke and ischemic heart disease (AHR=1.86; 95% CI: 1.27–2.73). Former and never smokers showed no significant differences.

**Table 2:** Association between smoking status and incidence of long-term care (LTC) certification: results from Cox proportional hazards models in the Yamagata Cohort Study, Yamagata, Japan, follow-up period January 2010–December 2020 (N=10430)

*Smoking status*	*Event*	*Unadjusted* *HR (95% CI)*	*p*	*Model 1* *AHR (95% CI)*	*p*	*Model 2* *AHR (95% CI)*	*p*	*Model 3* *AHR (95% CI)*	*p*
Current smokers (N=1291)	42	1.44 (1.03–2.02)	0.03*	1.81 (1.23–2.66)	<0.01*	1.83 (1.25–2.69)	<0.01*	1.86 (1.27–2.73)	<0.01*
Never smokers (ref.) (N=6700)	173								
Former smokers (N=2439)	47	0.90 (0.65–1.25)	0.54	0.81 (0.56–1.18)	0.28	0.81 (0.56–1.18)	0.27	0.79 (0.54–1.14)	0.21

Model 1: adjusted for sex and age. Model 2: adjusted as for Model 1 physical activity, alcohol consumption, hypertension, diabetes, and dyslipidemia. Model 3: adjusted as for Model 2 plus history of stroke and ischemic heart disease. AHR: adjusted hazard ratio.

* Statistically significant at p<0.05.

Cox proportional hazards model analysis results for the association between smoking status and LTC certification, stratified by sex and age groups, are presented in Table 3. In men, the unadjusted analysis indicated an HR for former smokers significantly lower than that for never smokers (HR=0.67; 95% CI: 0.45–1.00, p=0.05), with the upper CI limit reaching unity. However, no significant differences were observed in the adjusted models. In Model 1 (adjusted for age), the AHR for the smoker group was significantly higher than that for the never smoker group (AHR=1.52; 95% CI: 1.00–2.31, p=0.05). In Model 2 (adjusted for sex, age, physical activity, alcohol consumption, hypertension, diabetes mellitus, and dyslipidemia), the AHR for the smoker group was still significantly higher than that for the never smoker group (AHR=1.66; 95% CI: 1.09–2.54, p=0.02). In Model 3 (adjusted for sex, age, physical activity, alcohol consumption, hypertension, diabetes, dyslipidemia, stroke, and ischemic heart disease), the AHR for the smoker group was significantly higher than that for the never smoker group (AHR=1.70; 95% CI: 1.11–2.59, p=0.01).

**Table 3:** Association between smoking status and long-term care (LTC) certification stratified by sex in the Yamagata cohort study, Yamagata, Japan, follow-up period January 2010–December 2020 (N=10430)

*Smoking status*	*Event*	*Unadjusted* *HR (95% CI)*	*p*	*Model 1* *AHR (95% CI)*	*p*	*Model 2* *AHR (95% CI)*	*p*	*Model 3* *AHR (95% CI)*	*p*
**Men** (N=4587)									
Never smokers (ref.) (N=1490)	60								
Current smokers (N=1025)	36	1.12 (0.74–1.70)	0.58	1.52 (1.00–2.31)	0.05*	1.66 (1.09–2.54)	0.02*	1.70 (1.11–2.59)	0.01*
Former smokers (N=2072)	41	0.67 (0.45–1.00)	0.05*	0.69 (0.46–1.04)	0.08	0.72 (0.48–1.07)	0.11	0.72 (0.48–1.07)	0.10
**Women** (N=5843)									
Never smokers (ref.) (N=5210)	113								
Current smokers (N=266)	6	1.16 (0.51–2.64)	0.72	3.30 (1.44–7.58)	<0.01*	2.85 (1.22–6.63)	0.02*	2.83 (1.22–6.63)	0.02*
Former smokers (N=367)	6	0.94 (0.41–2.14)	0.88	1.80 (0.79–4.11)	0.16	1.60 (0.69–3.68)	0.27	1.53 (0.66–3.54)	0.32

Model 1: adjusted for age. Model 2: as for Model 1 plus physical activity, alcohol consumption, hypertension, diabetes, and dyslipidemia. Model 3: adjusted as for Model 2 plus history of stroke and ischemic heart disease. AHR: adjusted hazard ratio.

* Statistically significant at p<0.05.

The unadjusted analysis indicated no significant differences in women. However, in Model 1 (adjusted for age), the AHR for the smoker group was significantly higher than that for the never smoker group (AHR=3.30; 95% CI: 1.44–7.58, p<0.01). In Model 2 (adjusted for sex, age, physical activity, alcohol consumption, hypertension, diabetes, and dyslipidemia), the AHR for the smoker group was significantly higher than that for the never smoker group (AHR=2.85; 95% CI: 1.22–6.63, p=0.02). In Model 3 (adjusted for sex, age, physical activity, alcohol consumption, hypertension, diabetes, dyslipidemia, stroke, and ischemic heart disease), the risk of LTC certification remained significantly higher (AHR= 2.83; 95% CI: 1.22–6.63, p=0.02). Former and never smokers exhibited no significant differences in both men and women.

The results of sensitivity analysis with a Cox proportional hazards model that excluded individuals with a history of CVD (stroke or ischemic heart disease) are presented in Table 4. The significant AHR between smoking status and LTC certification was consistently maintained across all adjusted models.

**Table 4:** Association between smoking status and long-term care (LTC) certification after excluding participants with a history of cardiovascular disease in the Yamagata cohort study, Yamagata, Japan, follow-up period January 2010–December 2020 (N=10048)

*Smoking status*	*Event*	*Unadjusted* *HR (95% CI)*	*p*	*Model 1* *AHR (95% CI)*	*p*	*Model 2* *AHR (95% CI)*	*p*
Never smokers (ref.) (N=6517)	162						
Current smokers (N=1260)	40	1.46 (1.03–2.07)	0.03*	1.88 (1.26–2.80)	<0.01*	1.89 (1.27–2.81)	<0.01*
Former smokers (N=2271)	39	0.83 (0.59–1.18)	0.31	0.77 (0.51–1.15)	0.20	0.76 (0.51–1.14)	0.18

Model 1: adjusted for sex and age. Model 2: adjusted as for Model 1 plus physical activity, alcohol consumption, hypertension, diabetes, and dyslipidemia. AHR: adjusted hazard ratio.

* Statistically significant at p<0.05.

The results of the sensitivity analysis that excluded individuals with a history of CVD in the sex-based analysis are presented in Table 5. The unadjusted analysis revealed no significant difference; however, the significant AHR between smoking status and the occurrence of LTC certification was consistently maintained after adjustment.

**Table 5:** Association between smoking status and long-term care (LTC) certification stratified by sex after excluding participants with a history of cardiovascular disease in the Yamagata cohort study, Yamagata, Japan, follow-up period January 2010–December 2020 (N=10048)

*Smoking status*	*Event*	*Unadjusted* *HR (95% CI)*	*p*	*Model 1* *AHR (95% CI)*	*p*	*Model 2* *AHR (95% CI)*	*p*
**Men** (N=4339)							
Never smokers (ref.) (N=1424)	53						
Current smokers (N=997)	34	1.18 (0.77–1.82)	0.45	1.60 (1.03–2.47)	0.03*	1.73 (1.11–2.68)	0.02*
Former smokers (N=1918)	35	0.66 (0.43–1.02)	0.06	0.69 (0.45–1.06)	0.09	0.70 (0.46–1.08)	0.11
**Women** (N=5707)							
Never smokers (ref.) (N=5093)	109						
Current smokers (N=263)	6	1.19 (0.52–2.72)	0.67	3.42 (1.49–7.87)	<0.01*	2.91 (1.25–6.76)	0.01*
Former smokers (N=351)	4	0.66 (0.24–1.80)	0.42	1.33 (0.49–3.62)	0.58	1.17 (0.43–3.20)	0.77

Model 1: adjusted for age. Model 2: adjusted as for Model 1 plus physical activity, alcohol consumption, hypertension, diabetes, and dyslipidemia. AHR: adjusted hazard ratio.

* Statistically significant at p<0.05.

## DISCUSSION

This study analyzed the association between smoking status and LTC certification using Cox proportional hazards models. Current smoking was associated with an increased risk of LTC certification, even after adjustment for multiple confounders. Furthermore, this association persisted after excluding individuals with a history of CVD, suggesting that current smoking may be associated with a higher likelihood of LTC certification in Japan, independent of measured confounders.

Smoking prevalence in our cohort was lower than that reported in the general population based on the National Health and Nutrition Survey in Japan^26^. This discrepancy may be explained by the characteristics of our study population, which consisted of individuals undergoing health checkups. Such individuals are generally more health-conscious and more likely to engage in healthier behaviors, including lower smoking rates. Therefore, a ‘healthy volunteer’ bias^27,28^ may have contributed to the observed lower prevalence. This should be considered when interpreting the generalizability of our findings.

Smoking not only increases the risks of hypertension^7^, type 2 diabetes^10^, and CVDs^7,8^ but is also a risk factor for cerebrovascular disease^4^, a major cause of LTC certification^17^. Herein, smoking remained independently associated with LTC certification after adjustment for major diseases such as stroke and ischemic heart disease, suggesting the possibility of additional direct or unmeasured pathways underlying this association. Thus, in Japan’s aging society, smoking cessation may be associated with a reduced risk of requiring LTC in the future.

The AHR for former smokers did not significantly differ from that for never smokers. This result may indicate that the risk among former smokers could be comparable to that of never smokers, which aligns with the findings of prior studies^20-24^ reporting the effectiveness of smoking cessation in preventing the onset of a state requiring LTC and reaffirming its significance. Hence, smoking cessation contributes not only to improving the risk of developing smoking-related diseases and their prognosis but also to extending healthy life expectancy. Additionally, smoking cessation is expected to reduce the risk of requiring LTC.

In the sex-specific analysis, a significant increase in AHR was observed in the smoker group compared with the never smoker group for both men and women. Notably, the association appeared to be stronger in women than in men. Factors that may contribute to the stronger impact of smoking in women include selection bias due to low smoking rates, differences in baseline characteristics, biological susceptibility, and variations in health-related behavior. In addition, secondhand smoke exposure, which was not assessed in this study, may have differed by sex and could have acted as an unmeasured confounder, particularly among women with low active smoking prevalence. Previous studies comparing men and women with equivalent smoking histories have reported that women experience an earlier progression of smoking-related decline in lung function^29,30^ and have a higher risk of COPD-related hospitalization and mortality^31^, suggesting a potentially higher susceptibility to the effects of smoking in women than in men. Although the underlying mechanisms remain unclear, it has been reported that a mutation in the lung development-related gene CELSR1 is associated with COPD only in women^32^. Furthermore, differences in lung development^32^, metabolism^33^, and hormonal influences^34^ between the sexes may be implicated. Additionally, some reports have indicated that women have a higher risk of smoking-related ischemic heart disease^8,35^ than men, suggesting that the physical effects of smoking may be greater among female smokers.

Although the smoking rate among women in Japan is lower than that among men, an increase in smoking rates has been noted among women in their 40s to 60s^26^. As smoking raises concerns about the increased risk of requiring LTC, countermeasures that consider sex differences are necessary. However, caution is required in this interpretation owing to factors such as the small number of female smokers and significant differences in lifestyle habits among women, meaning that the distribution of confounding factors differs considerably by sex.

### Strengths and limitations

This study has several strengths, including its relatively large community-based sample and adjustment for multiple relevant covariates, including age, sex, physical activity, alcohol consumption, hypertension, and diabetes. However, several limitations should be acknowledged. First, the study population consisted of individuals who participated in routine health checkups in Yamagata Prefecture, representing a community-based convenience sample rather than a random or population-representative sample. Participants may have been more health-conscious than the general population, potentially introducing selection bias and limiting the generalizability of the findings. Consistent with this, the smoking prevalence observed in this study differed from that of the general Japanese population, suggesting that the study sample may not fully represent population-level smoking patterns. Second, smoking exposure was assessed using self-reported categorical data without biochemical validation and detailed information on smoking intensity, duration, cumulative exposure (e.g. pack-years), or time since cessation. Further, smoking status was based on participants’ self-identification rather than a standardized time-based or behavior-based definition (e.g. smoking within the past 30 days). This limitation is particularly important, as some individuals who actively smoke may not identify themselves as current smokers, potentially leading to exposure misclassification and underestimation of true smoking prevalence and its associations, as previously reported^36,37^. Third, smoking status and other lifestyle variables were assessed only at baseline and were not updated during the follow-up period. Therefore, changes in smoking behavior over time could not be captured, which may have led to additional misclassification and attenuation of associations. Fourth, alcohol consumption was categorized as current drinker versus non-drinker, and it was not possible to distinguish between lifetime abstainers and former drinkers. This may introduce bias, including the ‘sick quitter’ effect, whereby individuals who stop drinking due to health problems are misclassified as non-drinkers. Fifth, several covariates, including medical history variables such as stroke and ischemic heart disease, were based on self-reported questionnaire data and may be subject to recall bias and misclassification. In addition, the definition of dyslipidemia did not include LDL or HDL cholesterol due to data limitations, which may reduce comparability with standard clinical definitions. Sixth, although we adjusted for major cardiometabolic conditions, we could not account for other important potential confounders and mediators, including COPD, fractures, cognitive impairment, dementia, socioeconomic status, education level, occupation, and access to healthcare. As some of these factors are associated with both smoking and LTC risk, residual confounding may remain, and the observed associations may partly reflect indirect pathways. Seventh, information on secondhand smoke exposure was not available. This limitation may be particularly relevant for women and may have resulted in residual confounding or underestimation of the total effect of tobacco exposure. Eighth, although the maximum follow-up period was up to 10 years, the number of incident LTC certification events was relatively limited, which may have reduced statistical power and led to potential underestimation of long-term effects. Ninth, the outcome was defined using LTC certification data from the Japanese LTCI system. Although this system uses a standardized assessment process, the validity and completeness of registry data could not be fully evaluated. In addition, competing risks such as death were not explicitly accounted for, which may have influenced the estimated associations. Lastly, LTC certification reflects heterogeneous underlying causes of functional impairment. Therefore, the observed associations may vary depending on the specific conditions leading to disability. Despite these limitations, this study provides, to our knowledge, some of the first evidence from a Japanese community setting suggesting that smoking is associated with an increased risk of LTC certification, particularly among women. The findings suggest that smoking may be an important modifiable risk factor for LTC certification, with potential implications for functional decline and healthy life expectancy.

## CONCLUSIONS

This study found that current smokers had a higher risk of LTC certification than never smokers, suggesting that smoking may be an independent predictor of this condition. Furthermore, former smokers had a similar risk to never smokers.

## Supplementary Material



## Data Availability

The data supporting this study cannot be made available for privacy or ethical reasons, but are available from the corresponding author upon reasonable request after receiving permission from the Ethics Committee of Yamagata University.

## References

[1] Cabinet Office, Government of Japan. Annual report on the ageing society 2025; June, 2025. Accessed June 26, 2026. https://www8.cao.go.jp/kourei/english/annualreport/2025/pdf/2025.pdf

[2] Jin X, Mori T, Sato M, Watanabe T, Noguchi H, Tamiya N. Individual and regional determinants of long-term care expenditure in Japan: evidence from national long-term care claims. Eur J Public Health. 2020;30(5):873-878. doi:10.1093/eurpub/ckaa065 32556192 PMC7536255

[3] Olivares-Tirado P, Tamiya N, Kashiwagi M, Kashiwagi K. Predictors of the highest long-term care expenditures in Japan. BMC Health Serv Res. 2011;11:103. doi:10.1186/1472-6963-11-103 21575260 PMC3119177

[4] Yamada M, Arai H. Long-term care system in Japan. Ann Geriatr Med Res. 2020;24(3):174-180. doi:10.4235/agmr.20.0037 32829572 PMC7533196

[5] National Center for Chronic Disease Prevention and Health Promotion (US), Office on Smoking and Health. How tobacco smoke causes disease: the biology and behavioral basis for smoking-attributable disease: a report of the Surgeon General. Centers for Disease Control and Prevention (US); 2010. Accessed June 26, 2026. [Report in Japanese]. https://www.ncbi.nlm.nih.gov/books/NBK53017/21452462

[6] Ikeda N, Inoue M, Iso H, et al. Adult mortality attributable to preventable risk factors for non-communicable diseases and injuries in Japan: a comparative risk assessment. PLoS Med. 2012;9(1):e1001160. doi:10.1371/journal.pmed.1001160 22291576 PMC3265534

[7] Hozawa A, Okamura T, Murakami Y, et al. Joint impact of smoking and hypertension on cardiovascular disease and all-cause mortality in Japan: NIPPON DATA80, a 19-year follow-up. Hypertens Res. 2007;30(12):1169-1175. doi:10.1291/hypres.30.1169 18344621

[8] Iso H, Date C, Yamamoto A, et al. Smoking cessation and mortality from cardiovascular disease among Japanese men and women: the JACC study. Am J Epidemiol. 2005;161(2):170-179. doi:10.1093/aje/kwi027 15632267

[9] Fletcher C, Peto R. The natural history of chronic airflow obstruction. Br Med J. 1977;1(6077):1645-1648. doi:10.1136/bmj.1.6077.1645 871704 PMC1607732

[10] Willi C, Bodenmann P, Ghali WA, Faris PD, Cornuz J. Active smoking and the risk of type 2 diabetes: a systematic review and meta-analysis. JAMA. 2007;298(22):2654-2664. doi:10.1001/jama.298.22.2654 18073361

[11] Deal JA, Power MC, Palta P, et al. Relationship of cigarette smoking and time of quitting with incident dementia and cognitive decline. J Am Geriatr Soc. 2020;68(2):337-345. doi:10.1111/jgs.16228 31675113 PMC7002272

[12] Ohara T, Ninomiya T, Hata J, et al. Midlife and late-life smoking and risk of dementia in the community: the Hisayama study. J Am Geriatr Soc. 2015;63(11):2332-2339. doi:10.1111/jgs.13794 26503243

[13] Ward KD, Klesges RC. A meta-analysis of the effects of cigarette smoking on bone mineral density. Calcif Tissue Int. 2001;68(5):259-270. doi:10.1007/BF02390832 11683532 PMC5352985

[14] Kanis JA, Johnell O, Oden A, et al. Smoking and fracture risk: a meta-analysis. Osteoporos Int. 2005;16(2):155-162. doi:10.1007/s00198-004-1640-3 15175845

[15] Takashima N, Miura K, Hozawa A, et al. Cigarette smoking in middle age and a long-term risk of impaired activities of daily living: NIPPON DATA80. Nicotine Tob Res. 2010;12(9):944-949. doi:10.1093/ntr/ntq121 20675364

[16] Kojima G, Iliffe S, Walters K. Smoking as a predictor of frailty: a systematic review. BMC Geriatr. 2015;15:131. doi:10.1186/s12877-015-0134-9 26489757 PMC4618730

[17] Ministry of Health, Labour and Welfare (MHLW), Japan. 2022 (Reiwa 4) Nen kokumin seikatsu kiso chōsa no gaiyō [Comprehensive survey of living conditions in 2022]; 2022. Accessed June 26, 2026. [Report in Japanese]. https://www.mhlw.go.jp/toukei/saikin/hw/k-tyosa/k-tyosa22/index.html

[18] Tsuji I. Kenkō jūmyō oyobi chiiki kakusa no yōin bunseki to kenkō zōshin taisaku no kōka kenshō ni kansuru kenkyū [Research on factor analysis of healthy life expectancy and regional disparities and verification of the effects of health promotion measures]. Department of Public Health, Tohoku University Graduate School of Medicine; 2017. Accessed June 26, 2026. [Report in Japanese]. http://www.pbhealth.med.tohoku.ac.jp/pdf/kj-28.pdf

[19] Kitamura A, Seino S, Taniguchi Y, et al. Kōreisha no jiritsu sōshitsu ni oyobosu seikatsu shūkanbyō, kinōteki kenkō no renkan inshi no eikyō: Kusatsumachi kenkyū [Impact of lifestyle-related diseases and frailty on the incidence of loss of independence in Japanese community-dwelling older adults: a longitudinal study on aging and health in Kusatsu]. Nihon Koshu Eisei Zasshi. 2020;67(2):134-145. doi:10.11236/jph.67.2_134 32092729

[20] Hara M, Sobue T, Sasaki S, Tsugane S. Smoking and risk of premature death among middle-aged Japanese: ten-year follow-up of the Japan public health center-based prospective study on cancer and cardiovascular diseases (JPHC study) cohort I. Jpn J Cancer Res. 2002;93(1):6-14. doi:10.1111/j.1349-7006.2002.tb01194.x 11802802 PMC5926871

[21] Duncan MS, Freiberg MS, Greevy RA Jr, Kundu S, Vasan RS, Tindle HA. Association of smoking cessation with subsequent risk of cardiovascular disease. JAMA. 2019;322(7):642-650. doi:10.1001/jama.2019.10298 31429895 PMC6704757

[22] Bullen C. Impact of tobacco smoking and smoking cessation on cardiovascular risk and disease. Expert Rev Cardiovasc Ther. 2008;6(6):883-895. doi:10.1586/14779072.6.6.883 18570625

[23] Rigotti NA, Clair C. Managing tobacco use: the neglected cardiovascular disease risk factor. Eur Heart J. 2013;34(42):3259-3267. doi:10.1093/eurheartj/eht352 24014389

[24] Anthonisen NR, Skeans MA, Wise RA, et al. The effects of a smoking cessation intervention on 14.5-year mortality: a randomized clinical trial. Ann Intern Med. 2005;142(4):233-239. doi:10.7326/0003-4819-142-4-200502150-00005 15710956

[25] Kashiwagi A, Kasuga M, Araki E, et al. International clinical harmonization of glycated hemoglobin in Japan: from Japan diabetes society to national glycohemoglobin standardization program values. J Diabetes Investig. 2012;3(1):39-40. doi:10.1111/j.2040-1124.2012.00207.x PMC4014931 24843544

[26] Kokumin kenkō eiyō chōsa [National health and nutrition survey]. Ministry of Health, Labour and Welfare, Japan. Accessed June 26, 2026. [Website in Japanese]. https://www.mhlw.go.jp/bunya/kenkou/kenkou_eiyou_chousa.html

[27] Thomson CA, Harris RB, Craft NE, Hakim IA. A cross-sectional analysis demonstrated the healthy volunteer effect in smokers. J Clin Epidemiol. 2005;58(4):378-382. doi:10.1016/j.jclinepi.2004.10.013 15868696

[28] Fry A, Littlejohns TJ, Sudlow C, et al. Comparison of sociodemographic and health-related characteristics of UK Biobank participants with those of the general population. Am J Epidemiol. 2017;186(9):1026-1034. doi:10.1093/aje/kwx246 28641372 PMC5860371

[29] Amaral AFS, Strachan DP, Burney PGJ, Jarvis DL. Female smokers are at greater risk of airflow obstruction than male smokers. UK Biobank. Am J Respir Crit Care Med. 2017;195(9):1226-1235. doi:10.1164/rccm.201608-1545OC 28075609

[30] Sørheim IC, Johannessen A, Gulsvik A, Bakke PS, Silverman EK, DeMeo DL. Gender differences in COPD: are women more susceptible to smoking effects than men?. Thorax. 2010;65(6):480-485. doi:10.1136/thx.2009.122002 20522842 PMC8191512

[31] Prescott E, Bjerg AM, Andersen PK, Lange P, Vestbo J. Gender difference in smoking effects on lung function and risk of hospitalization for COPD: results from a Danish longitudinal population study. Eur Respir J. 1997;10(4):822-827. 9150319

[32] Hardin M, Cho MH, Sharma S, et al. Sex-based genetic association study identifies CELSR1 as a possible chronic obstructive pulmonary disease risk locus among women. Am J Respir Cell Mol Biol. 2017;56(3):332-341. doi:10.1165/rcmb.2016-0172OC 27854507 PMC5359539

[33] Ben-Zaken Cohen S, Paré PD, Man SF, Sin DD. The growing burden of chronic obstructive pulmonary disease and lung cancer in women: examining sex differences in cigarette smoke metabolism. Am J Respir Crit Care Med. 2007;176(2):113-120. doi:10.1164/rccm.200611-1655PP 17413125

[34] Peng J, Xu X, Mace BE, et al. Estrogen metabolism within the lung and its modulation by tobacco smoke. Carcinogenesis. 2013;34(4):909-915. doi:10.1093/carcin/bgs402 23276798 PMC3616670

[35] Huxley RR, Woodward M. Cigarette smoking as a risk factor for coronary heart disease in women compared with men: a systematic review and meta-analysis of prospective cohort studies. Lancet. 2011;378(9799):1297-1305. doi:10.1016/S0140-6736(11)60781-2 21839503

[36] Leas EC, Zablocki RW, Edland SD, Al-Delaimy WK. Smokers who report smoking but do not consider themselves smokers: a phenomenon in need of further attention. Tob Control. 2015;24(4):400-403. doi:10.1136/tobaccocontrol-2013-051400 24500273

[37] Connor Gorber S, Schofield-Hurwitz S, Hardt J, Levasseur G, Tremblay M. The accuracy of self-reported smoking: a systematic review of the relationship between self-reported and cotinine-assessed smoking status. Nicotine Tob Res. 2009;11(1):12-24. doi:10.1093/ntr/ntn010 19246437

